# Limitations and Potential Clinical Application on Contrast Echocardiography

**DOI:** 10.2174/157340310790231653

**Published:** 2010-02

**Authors:** Elisa Modonesi, Manrico Balbi, Gian Paolo Bezante

**Affiliations:** Department of Cardionephrology and Department of Internal Medicine, University of Genoa, Genoa, Italy

**Keywords:** Contrast echocardiography, myocardial perfusion.

## Abstract

Myocardial contrast echocardiography (MCE) is a relatively simple myocardial perfusion imaging technique which should be used in different clinical settings. The ability of MCE to provide a comprehensive assessment of cardiac structure, function, and perfusion is likely to make it the technique of choice for non-invasive cardiac imaging.

Contrast agents are encapsulated microbubbles (MB) filled with either air or high-molecular-weight gas. They are innocuous, biologically inert and when administered intravasculary, the sound backscatter from the blood poll is enhanced because MB have the enormous reflective ability due to a large acoustic impedance mismatch between the bubble gas and surrounding blood.

MCE is an ideal imaging tool for the assessment of left heart contrast and the myocardial microcirculation. MCE detects contrast MB at the capillary level within the myocardium and, thus, has the potential to assess tissue viability and the duration of the contrast effect. MCE was equivalent to SPECT for the detection of CAD with a tendency toward higher sensitivity of MCE compared with SPECT in microvascular disease and CAD. MCE is also a bedside technique that can be used early in patients presenting with acute heart failure to rapidly assess LV function (regional and global) and perfusion (rest and stress).

The MB are smaller than the capillaries and should flow freely through the coronary circulation. It is important to remember that the actual risk associated with contrast is very small, especially if physician follow the contrast administration recommendations that suggest avoiding the use of MB in unstable and critically acute ill patients.

## PHYSICAL CHARACTERISTICS OF CONTRAST AGENT

Contrast agents are encapsulated microbubbles (MB) filled with either air or highmolecular-weight gas. They are innocuous, biologically inert and when administered intravasculary, the sound backscatter from the blood poll is enhanced (non-linear acoustic behaviour) because MB have the enormous reflective ability due to a large acoustic impedence mismatch between the bubble gas contenent and surrounding blood.

The ability of MB to produce strong backscattered acoustic signal is based on their compressibility, which depends on the viscoelastic and pressure properties of the shell and gas. MB encapsulation is required to increase stability and persistence, which depends on rigidity and provides persistence and good resistance to blood pressure changes and US pressure waves. A degree of fragility is needed to allow the destruction and posterior myocardial refilling. Another approaches to extend MB survival are the use of gas with low solubility and diffusibility.

The diameter of MB is one of the major determinants of scattering behavior because the scattering cross section is extremely dependent on the bubble size and is directly proportional to the sixth power of its radious [1]. Larger MB produces higher US signal and any reduction in the size results in a marked decrease of its reflection ability. The range diameter is from 1 to 10 µm, and the ideal size is approximately 4 µm (Table **1**). The signal intensity depends on MB concentration: at low concentration the signal intensity increase linearly; as the concentration increases, the relationship between MB and signal intensity becomes curvilinear until it reach a plateau, where the contrast effect may actually decrease.

The interaction between MB and US produces compression during pressure peaks and expansion during pressure nadirs. It appears a remarkable and fortuitous coincidence that gas bubbles of a size required to cross the pulmonary capillary vascular resonate in a frequency range of 1.5–7 MHz, precisely that used in diagnostic US echocardiography. The oscillation of MB may produce a wide range of harmonic frequencies: subharmonic [2, 3], second harmonic, ultraharmonics [4] and super harmonics (third, fourth, fifth). The harmonic response is dependent upon the physical characteristics of the agent (both size and mechanical properties), the incident pressure of the US field, and the frequency [5]. In particular, the responce of MB depend also about their mechanical index (MI). MI is defined as the peak of negative pressure divided by square root of the US frequency. Unlike solid tissue, gas bubbles have acoustic properties that vary with the strength of the insonating signal. With increasing power, insonation of gas bubbles can result in linear oscillation (MI<0,1), non-linear oscillation (MI 0,1-1,0) or bubble destruction (MI>1,0 scintillation) [6, 7].

## CLASSIFICATION

The first agents capable of left heart contrast after intravenous injection (first generation agents) were air bubbles stabilised by encapsulation (Albunex)^®^ or by adherence to microparticles (Levovist)^®^. Replacing air with a low solubility fluorocarbon gas stabilises bubbles still further (second generation agents—for example, Optison, SonoVue)^®^, greatly increasing the duration of the contrast effect. Third generation agents—not yet commercially available—will use polymer shell and low solubility gas and should produce much more reproducible acoustic properties. These properties allow passage of the pulmonary vasculature, opacification of the left ventricular cavity, and imaging of myocardial perfusion. Currently, the most used second-generation contrast agents are Sonovue^®^ (Bracco, Milan, Italy), Optison^®^ (Amersham Health AS, Oslo, Norway), and Definity^®^ (Bristol-Myers Squibb, Billerica, Massachusetts) (Table **1**). These agents differ in terms of their shell constituents and gas content that influence shell-stiffness and stability of the MB, and determine physical properties [8]. Even if these agents are licensed for left ventricular opacification only, all are suitable for myocardial perfusion.

## IMAGING MODALITIES TO ASSESS MICROCIRCULATION

Myocardial contrast echocardiography (MCE) is an ideal imaging tool for the assessment of the myocardial microcirculation. MCE detects contrast MB at the capillary level within the myocardium and, thus, has the potential to assess tissue viability. At baseline, approximately 8% of left ventricular mass is constituted by blood present in the microcirculation termed myocardial blood volume (MBV), 90% of which is comprised of blood in the capillaries. In normally perfused myocardium, the rate of capillary blood flow is 1 mm/s. Saturation of the coronary microvasculature by MB therefore takes about 5 s (because the thickness of the US beam is approximately 5 mm). When there is no flow limiting stenosis, MBF increases 5 times during hyperemia (stress testing), and therefore the myocardium replenishes in 1 s. In addition, a flow-limiting stenosis leads to a reduction in capillary blood volume in the distal microvasculature, with an accompanying decrease in signal intensity during MCE. These 2 features (slowed contrast appearance and decreased capillary blood volume) form the basis for detecting CAD using MCE. However, because the US beam used to detect the MB also destroys them at normal power outputs, the lower the MBF the longer the time required to achieve peak video intensity.

The mechanism of US images enhancement depends on MB’behavior under acoustic pressure. The enormous reflective ability of MB is due to a large acoustic impedence mismatch between the bubble gas content and the surrounding blood. In contrast to MB, cardiac tissue produces much fewer harmonic frequencies, so selective reception of harmonic echos will preferentially detect signals emanating from contrast agent rather than the myocardium. Although these methods improved the signal-to-noise ratio, off-line image processing is frequently required to evaluate myocardial perfusion, because tissue signals is still present. Harmonic imaging also used high acoustic powers that destroyed MB consequently, the imaging frame rate had to be reduced substantially with electrocardiographic triggering to allow MB to replenish the myocardial microcirculation between pulses (Table **2**).

These include high-power modalities with superior tissue noise suppression that allow on-line assessment of myocardial perfusion and low-power modalities that permit imaging with high frame rates. These modalities specifically take advantage of the nonlinear behavior of MB in an acoustic field. Many techniques use multiple transmitted pulses that are, e.g. full and half amplitude (power modulation imaging, Fig. (**1**). to distinguish nonlinear MB signals from tissue. Reflected echos are scaled and subtracted. Because tissue responds linearly (especially at low acoustic powers), subtraction and scaling results in zero signal. MB reflect nonlinearly, so received echos will not be canceled out, enabling selective MB detection. Most of these multipulse techniques additionally use power Doppler, and the resulting signal is color coded and displayed. Because high-power imaging destroys MB, imaging of myocardial perfusion cannot be performed in real time. Low MI imaging increases signal-to-noise ratio and because of minimal MB destruction continuous imaging may be performed. Both high- and low-power MCE have their advantages and disadvantages. High-power imaging precludes continuous imaging owing to destruction of contrast, and therefore wall motion cannot be assessed simultaneously. On the other hand, signal-to-noise ratio is good. Low-power imaging allows simultaneous assessment of perfusion and contraction but has a lower sensitivity for the detection of MB.

## MYOCARDIAL PERFUSION: DEFINITION AND RILEVANCE

Myocardial perfusion imaging is a functional cardiac imaging, used for the diagnosis of ischemic heart disease [9]. The underlying principle is that under conditions of stress, diseased myocardium receives less blood flow than normal myocardium. This method needs a inert myocardial tracer, that injected in a peripheral vein, is able to reach the coronary vascular bed and a cardiac imaging system devoted to detect normal or abnormal myocardial distribution of the tracer during rest and stress conditions. Following this, the heart rate is raised to induce myocardial stress, either by exercise or pharmacologically with adenosine, dobutamine or dipyridamole (aminophylline can be used to reverse the effects of dipyridamole). Imaging techniques performed after stress reveals the distribution of the tracer, and therefore the relative blood flow to the different regions of the myocardium. Diagnosis is made by comparing stress images to a further set of images obtained at rest. Nuclear myocardial perfusion studies are the most utilized imaging techniques to evaluate myocardial perfusion, while an image technique as MCE that could simultaneously detect wall motion and measure perfusion was indeed the holy grail of coronary artery disease (CAD) testing is still potential toll under evluation mainly due to safety issues. Contrast echocardiography is a potential technique to evaluate myocardial perfusion. A cardiac specific radiopharmaceu-tical (E.g. 99mTc-tetrofosmin-Myoview®, GE healthcare, 99mTc-sestamibi-Cardiolite®, Bristol-Myers Squibb) or a micro-bubble contrast agents like Optison®, Definity® or SonoVue® should be administered.

## SAFETY OF CONTRAST AGENT

MB can potentially affect the heart by mechanical obstruction of the coronary vessels or cause direct cell damage when destroyed in high-intensity sound fields. Mechanical obstruction can occur when bubbles larger than capillary diameter are injected into the coronary circulation. However, the bubbles are generally smaller than the capillaries and should flow freely through the coronary circulation. Even without a specific mechanism, the fatal aderse events in unstable patients suggest that increased caution is appropriate. First, it is important to remember that the actual risk associated with contrast is very small. Second, the new list of contraindications for encapsulated MB contrast agents (Table **3**) would preclude most applications in the intensive care unit/critical care unit. Likewise the recommendation that patients be monitored for 0.5 h after injection will limit use in portable studies on nonmonitored hospitalized patients and, in many laboratories will complicate outpatient studies. This would only require extension of the postprocedure monitoring [10, 11]. These new warnings also raise the issue of informed consent.

While there is no doubt that the use of contrast enables acquisition of US images of improved quality, these setbacks in contrast agent safety are not the only problem. There has been slower than expected adoption of their use, whether due to low reimbursement or the "hassle factor" of an intravenous medication, and no agent has yet been approved for myocardial perfusion. In the end, these new warnings do not preclude the use of contrast, particularly in situations where the information is important and is either not available in any other way or could only be obtained with tests that have their own inherent complications.

## RELATION BETWEEN MCE AND SPECT PERFUSION

From the blood pool, 99mTc-sestamibi diffuses into the extravascular space and passively enters the myocyte before binding to the negatively charged mitochondrial membrane. Its retention within the myocyte, therefore, is dependent on intact mitochondrial function. Up to about two times normal flow, the myocardial uptake of 99mTc-sestamibi during exogenously induced hyperemia is determined by flow to that region. The relative distribution of 99mTc-sestamibi in various myocardial beds, therefore, reflects differential blood flow to those beds. Consequently, the presence of a perfusion mismatch during hyperemia indicates the presence of physiologically significant coronary stenosis. During rest, 99mTc-sestamibi imaging provides information not only on baseline flow but also myocyte viability. Necrotic areas do not retain 99mTc-sestamibi and demonstrate reduced uptake. In comparison, absent or reduced contrast enhancement is seen within necrotic regions on MCE because of reduced myocardial blood volume consequent to microvascular disruption, plugging, and obliteration [12]. Regions with these patterns have been shown to demonstrate lack of recovery of function despite the presence of open infarct-related arteries [13].

## MYOCARDIAL CONTRAST ECHOCARDIOGRAPHY AS A TECHNIQUE OF MYOCARDIAL PERFUSION

Unlike other tracers used for assessing myocardial perfusion, MB used during MCE reside entirely within the vascular space. They do not enter the extravascular space or are extracted by myocytes. After peripheral injection, MB mix with blood in the central circulation, and their concentration at any given time within a myocardial region of interest reflects the volume of blood within that region. When changes in myocardial blood flow (MBF) are regulated by changes in MBV (as occurs with dipyridamole, which causes vasodilation of the coronary microcirculation), relative US intensity ratios within different myocardial regions reflect relative ratios of both MBV and MBF in these regions. It is well known that in the presence of a stenosis, MBF remains constant until the stenosis is severe (> 85% of luminal diameter narrowing). MBF is maintained by vasodilation and/or recruitment of <300-μm microvessels distal to the stenosis. Thus, it is accepted that at rest, MBV increases within the bed supplied by a stenotic artery. Because it causes vasodilation of all microvessels, it is also generally assumed that MBV is equal between beds supplied by stenosed and nonstenosed vessels during exogenous hyperemia. Since during exogenous hyperemia, MBV and flow are closely coupled, the ratios of video intensities from different beds have been demonstrated to reflect the ratios of MBF to those beds.

## STRESS PROTOCOL IN CLINICAL PRACTICE

Stress agent. The most commonly used stress agents are adenosine, dipyridamole, and dobutamine (Table **4**). All 3 agents have been widely used for pharmacologic stress-TESTS [14-17]. Although different working mechanism, all agents have a high diagnostic value for detection of CAD. It is well known that MCE can define the presence of abnormal perfusion at rest and during pharmacologic stress in stable CAD subjects, with a high concordance between MCE and 99mTc-sestamibi single photon emission computerized tomography (SPECT). Also, comparison of accelerated intermittent imaging at rest and with exercise to SPECT demonstrated a concordance of 76% to 92%. Several other studies assessed the accuracy of MCE and SPECT/dobutamine stress echocardiography for detection of stable CAD. In general, there is a good agreement between techniques, ranging from 65% to 92%. Furthermore, the addition of MCE may improve sensitivity for detection of CAD over wall motion analysis during stress. MCE provides prognostic value in patients with stable CAD. Patients with normal perfusion have a better outcome than patients with normal wall motion, underscoring the value of incorporating MCE in stress echocardiography.

## MCE IN THE ISCHEMIC SYNDROMES

Currently, the diagnosis of acute ischemic syndromes (ACS) is based on the triad of clinical history, electrocardiography, and laboratory investigation. These methods, although useful, can often be nondiagnostic in this setting. Because MCE is the only technique that permits immediate assessment of wall motion and perfusion, it has a unique role in the diagnosis of ACS [18]. Myocardial contrast echocardiography allows quick evaluation of myocardial perfusion in the emergency department and may be used to triage patients into a low- or high-risk category. The value of MCE in ACS has been studied experimentally using coronary balloon occlusions in pigs. MCE accurately reflects the decrease in myocardial perfusion during balloon occlusion compared with microsphere-derived MBF. Studies have also shown that during coronary occlusion the area at risk correlates closely with the contrast defect early after contrast flash destruction and that the plateau contrast defect identifies infarct size. Kamp *et al*. [19] were the first to report the sensitivity of MCE to detect perfusion defects in patients suspected of having acute myocardial infarction (AMI). With 1:1 end-systolic–triggered imaging, MCE perfusion defects were detected in 19 of 32 patients (59%) with Thrombolysis In Myocardial Infarction (TIMI) flow grade 0 before percutaneous transluminal coronary angioplasty. The sensitivity of MCE tended to decrease when patients had better TIMI flow and inferior infarctions (20%), whereas the sensitivity of MCE in patients with an anterior coronary artery occlusion Is high (88%). Other studies assessing the potential of MCE to ACS also reported high sensitivities, comparable with those of standard echocardiography and SPECT. In addition, MCE appears to have important prognostic value in patients presenting to the emergency department with acute chest pain [20]. Besides detection of acute ischemic heart disease, MCE may play a pivotal role in prediction of functional recovery in patients after ST-segment elevation myocardial infarction. The severity of myocardial damage is currently mainly estimated by enzymatic damage and wall motion score, whereas recovery of myocardial function is importantly dependent on reflow of blood to the risk area [21]. The presence of reflow after coronary angioplasty is suggested by resolution of chest pain and the degree of resolution of ST-segment elevation but can be visualized by MCE. Kloner [22] was the first to demonstrate the deleterious effect of no-reflow on clinical outcome. Later, it was shown that the presence of no-reflow on MCE after AMI related to absence of preinfarction angina, number of Q waves, wall motion score at presentation, TIMI flow grade 0, the size of the area at risk, and the occlusion status of the culprit artery [23]. Conversely, intact microvasculature after AMI (reflow), is a positive predictor of functional recovery [24]. Patients without microvascular dysfunction on MCE have less enzymatic elevation, better functional performance, better recovery of global and regional wall motion, less remodeling, and better survival independent of other predictors [25, 26].

The ability of MCE to predict functional recovery is comparable to that of cardiovascular magnetic resonance imaging [27]. Thus the existing literature suggests that MCE has important additional value for diagnosis and risk stratification in patients with acute ischemic heart disease. The presence of a region of no-reflow is important in the decision of therapeutic strategy to predict the severity and duration of left ventricular dysfunction in the early stage of an myocardial infarction. MCE with no reflow detect clinical congestive heart failure in the early phase of the necrotic event. The higher frequency of early congestive heart failure in patients with no-reflow is still not clear. It seems that the no reflow phenomenon, the culprit lesion reflecting the size of the risk area, and age are related to early congestive heart failure [28]. The main reason of standing heart failure seems due to the larger area of infarction in patients with MCE no reflow than in those with MCE reflow; thus, it may take longer for the left ventricle to adapt to a larger infarction.

## MCE TO DETECT TARGET ORGAN DAMAGE

The study by Aggeli *et al.* [29] demonstrated the value of exercise test is the most widely used technique for the assessment of CAD, but it has a relatively low sensitivity and specificity compared with imaging techniques such as stressechocardiography SPECT imaging. Systemic hypertension is a strong risk factor for CAD, but the prevalence of CAD in such patients is moderate.

An exercise test is the most widely used technique for the assessment of CAD, but it has a relatively low sensitivity and specificity compared with imaging techniques such as stress echocardiography and SPECT imaging.

Stress echocardiography is excellent for the assessment of CAD, but its sensitivity tends to be compromised in patients with significant left ventricular hypertrophy (LVH). Perfusion techniques like SPECT may suffer from low specificity, because hypertensive patients may have microvascular disease in the absence of large vessel CAD and often asymetrical LVH, which result in a relative difference in tracer uptake leading to apparent perfusion abnormalities even in the absence of microvascular disease and CAD. The MCE in this scenario is an ideal technique [30]. Perfusion defect in the presence of vasodilator stress almost always occurs when there is significant epicardial coronary stenosis. Microvascular disease is manifested as delayed MB filling of the myocardium after a destructive phase. These differential effects on the kinetics of MCE and differential manifestation as a result may allow MCE to accurately classify patients with and without CAD as opposed to microvascular disease. In the study by Aggeli *et al*. MCE was equivalent to SPECT for the detection of CAD with a tendency toward higher sensitivity of MCE compared with SPECT. The latter is likely because MCE, by virtue of its superior spatial resolution compared with SPECT, is likely to identify mild subendocardial ischemia due to CAD more effectively.

## MCE IN HEART FAILURE

MCE is a bedside technique that can be used early in patients presenting with acute heart failure (AHF) to rapidly assess LV function (regional and global) and perfusion (rest and stress) [31]. Demonstration of normal resting myocardial perfusion in patients with AHF indicates viable myocardium [32]. The presence of significant reversible myocardial perfusion defects in these patients establishes the diagnosis of flow-limiting CAD and should prompt the attending physician to plan for urgent coronary arteriography and revascularization [33]. Coronary arteriography may not be warranted in patients with no demonstrable myocardial perfusion defects. However, even in the absence of obvious myocardial perfusion defects, determination of MBF reserve may help to predict the outcome of patients with heart failure. These findings are likely to affect the routine practice of all AHF patients in our institution undergoing coronary arteriography.

## CONCLUSIONS

Myocardial contrast echocardiography is a relatively simple technique for imaging of myocardial perfusion that should be used in different clinical settings. The ability of MCE to provide a comprehensive assessment of cardiac structure, function, and perfusion is likely to make it the technique of choice for non-invasive cardiac imaging. MCE is accurate in distinguishing different patterns of myocardial perfusion suggesting a potential risk-stratification technique in subjects without a prior history of CAD or clinical features suggestive of an ACS.

## Figures and Tables

**Fig. (1):**
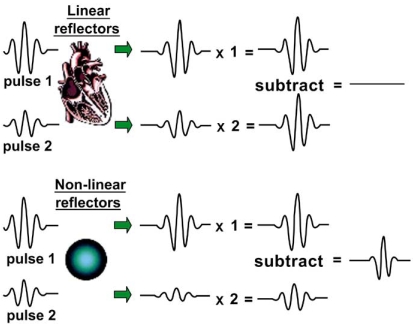
Power Modulation Imaging. In Power Modulation Imaging two consecutive pulses with identical shape but a twofold difference in amplitude are sent out and result in identical reflections from cardiac tissue, other than the expected twofold difference in amplitude. The smaller pulse is then multiplied by two and subtracted from the larger one, resulting in zero signal. The same two pulses, when reflected by nonlinear MB, produce signals of different shape and amplitude. Amplifying the smaller pulse and subtracting it results in a non-zero signal.

**Table 1.** Principal Parameters of MCE

	Definity	Optison	Sonovue
Composition	Perflutren lipid microsphere	Perflutren protein-type A	Sulphur hexafluoride in form of MB
Company	Bristol-Myers Squibb	Amersham Health AS	Bracco Imaging
Shell composition	Lipid	Albumin	Lipid- no shell surfactant stabilized
Mean diameter (µm)	1,1-3,3	3,0-4,5	2,5
Range	% less than 10 µm is 98%; maximum diameter 20 µm	% less than 10 µm is 98%; maximum diameter 32 µm	% less than 11 µm 99%; maximum diameter 20 µm
Metabolism	Stable gas not metabolized. Phospholipid component are metabolized to free fatty acids.	Stable gas not metabolized. Albumin handled by the normal metabolic route.	Sulphur exafluoride dissolves in the blood and is subsequently exhaled.
Elimination	By lungs; not detectable in the blood or expider air after 10’	By lungs; not detectable in the blood or expider air after 10’	By lungs; not detectable in the blood or expider air after 15’
Bolus	10 mL/Kg iv bolus injection whitin 30-60sec, followed by 10mL of saline flush.	0.5 mL iv. This may be repeated for further contrast enhancement.	2 or 2.4 ml and might be repeated. Every injection should be followed by saline flush.
Infusion	1,3 mL added to 50 mL saline; rate of infusion should be initiated at 4.0 mL/min, but titraded as necessary	The injection rate should not exceed 1 mL per second, followed by a saline flush.	at 50 to 70 mL/h with VueJect (BR-INF 100,Bracco Research SA)
Maximun dosage	Not exceed 10 mL/min. The maximum dose is either low bolus doses or one single iv infusion.	Not exceed 5.0 mL in any 10 minute period. The maximum total dose should not exceed 8.7 mL in any one patient study.	In a Phase I study doses up to 56 ml of SonoVue were administered to normal volunteers without serious adverse events being reported.

**Table 2.** Contrast Specific Imaging Methods for Assessment of Myocardial Perfusion

	Harmonic B-mode	Harmonic power Doppler	Pulse invertion	Power pulse invertion
Bubble-to-tissue sensivity	Moderate	Very good	Good	Very good
Off-line background subtraction needed	Yes	No	Yes	No
LV-myocardium delineation	Poor	Good	Moderate	Good
Wall motion artifacts	None	Can be severe	Moderate	Few
Real-time imaging	No	No	No	Yes

**Table 3.** Contraindications to the Use of Optison, Definity or Sonovue

- Intra-arterial injection
- Right-to-left, bi-directional, or transient right- to-left cardiac shunts
- Worsening or clinically unstable congestive heart failure
- Acutemyocardial infarction or acute coronary syndromes
- Ventricular arrhythmias or high risk for arrhythmias due to QT prolongation
- Respiratory failure, as manifest by signs or symptoms of carbon dioxide retention or hypoxemia
- Severe emphysema, pulmonary emboli or other conditions that cause pulmonary hypertension due to compromised pulmonary arterial vasculature
- Hypersensitivity to perflutren, blood, blood products, or albumin.

**Table 4:** Stress Protocol in Clinical Practice

	HR	BP	Scan	Regional and global function	
				*Normal response*	*Ischemic response*
Dobutamine	Increase	Increase systolic/diastolic BP	Difficult	Increase function and velocity of contraction compared with rest and usually with low dose; Greater decrease in ESV, marked increase in EF	Decrease function and velocity of contraction compared with low dose; may be less compared with rest; Often same as normal response, rarely, ischemia produces decreased EF; cavity dilatation rarely occurs
Vasodilator: Dipyridamole/Adenosine	Reduction	Decrease systolic/diastolic BP	Easier	Increase function compared with rest; Decrease in ESV, increase in EF	Decreased function compared with rest; Often same as normal response; occasionally, ischemia produces decreased EF; cavity dilatation rarely occours.

## ABBREVIATION

ACS: = Acute coronary sindrome
AHF: = Acute heart failure
AMI: = Acute myocardial infarction
CAD: = Coronary artery disease
LVH: = Left ventricular hypertrophy
MB: = Microbubbles
MBF: = Mycardial blood flow
MBV: = Mycardial blood volume
MCE: = Mycardial contrast echocardiography
MI: = Mechanical index
SPECT: = Single photon emission computerized tomography
US: = Ultrasound
